# Physical activity and sleep relate to antibody maintenance following naturalistic infection and/or vaccination in older adults

**DOI:** 10.1016/j.bbih.2023.100661

**Published:** 2023-06-25

**Authors:** Anna C. Whittaker, Len De Nys, Ryan C. Brindle, Mark T. Drayson

**Affiliations:** a Faculty of Health Sciences and Sport, University of Stirling, UK; b Department of Cognitive and Behavioural Science & Neuroscience Program, Washington and Lee University, USA; c Institute of Immunology and Immunotherapy, University of Birmingham, UK

**Keywords:** ageing, Antibodies, Naturalistic immunity, Physical activity, Sleep, Vaccination

## Abstract

Health behaviours such as being physically active and having good quality sleep have been associated with decreased susceptibility to infection and stronger antibody responses to vaccination. Less is known about how such factors might influence the maintenance of immunity following naturalistic infection and/or prior vaccination, particularly among older adults who may have formed initial antibodies some time ago. This analysis explored antibody levels against a range of common infectious diseases in 104 older adults (60 women) aged 65+ years, and whether these relate to self-reported physical activity (PA) and sleep. PA and sleep were measured subjectively through standardized questions. Antibody levels to a range of common pathogens, including pneumococcal (Pn) and meningococcal (Men) serotypes, *Haemophilus influenza* type b, diphtheria, and tetanus were assayed using Multiplex technology. Higher PA at baseline related to higher antibody levels against three Pn serotypes and MenY, and higher PA at one month with higher levels against six Pn serotypes. Longer time in bed related to higher antibody levels against Pn4, and longer sleep related to higher levels against Pn19f. More difficulty staying awake in the day related to lower antibodies against Pn19a, Pn19f, MenA and MenY, and more frequent daytime napping related to lower levels against three Pn serotypes and MenY. Using clinically protective antibody thresholds as an outcome showed similar results for PA, but effects for sleep became non-significant, with the exception of time in bed. This extends beyond existing literature demonstrating associations between PA and sleep and peak antibody response to vaccination to antibody maintenance. Longitudinal research with objective measures of health behaviours is warranted.

## Highlights

• Physical activity relates to maintaining immunity to common infectious diseases.
• Higher PA levels relate to higher antibodies against pneumococcal serotypes.
• Longer time in bed and sleep, and less daytime napping relates to higher antibodies.
• Associations between PA, sleep and antibody maintenance differs by pathogen type.

## 1 Introduction

Measuring the antibody response to vaccination has been used widely as a useful means of studying psychosocial and behavioural influences on health via effects on *in vivo* immune function (Kiecolt-Glaser et al., 2021; Phillips, 2011). Identifying variables that affect antibody responses to vaccination is important to improve immunity and, therefore, health, especially if those identified are modifiable factors such as health behaviours. Health behaviours, including physical activity (PA), diet, sleep, tobacco, alcohol, or substance use, are well known to influence health and wellbeing via many different physiological mechanisms and are regularly addressed in public health campaigns and medical advice, e.g., (“Live Well - NHS,” n.d.). Such behaviours are also known to impact immunity and the antibody response to vaccination, including Covid-19 vaccination, e.g. (Chastin et al., 2021; McElhaney, 2002), and can be targeted through interventions to improve vaccination immunogenicity and efficacy (Zimmermann and Curtis, 2019).

There is considerable research on the impact of PA on the antibody response to vaccination. For example, studies have shown that higher levels of habitual 10.13039/100006131PA relate to a stronger antibody response to common thymus-dependent vaccinations like influenza in young (Whitham and Blannin, 2003) and older adults (de Araújo et al., 2015), partly supported by a recent systematic review (Bohn-Goldbaum et al., 2022). Further, exercise interventions have been related to stronger vaccination responses in several training trials where participants receive an intervention to increase their PA levels compared to control groups, e.g., (Felismino et al., 2021; Kohut et al., 2004, 2005, Schuler et al., 2003, Woods et al., 2009), although not all interventions have successfully improved vaccination responses, e.g., (Hayney et al., 2014; Long et al., 2013).

Similarly, sleep has also been examined as a health behaviour that can influence immunity, and specifically the antibody response to vaccination (Rayatdoost et al., 2022). Such studies have shown that both sleep duration and sleep quality have the potential to impact antibody responses, (Taylor et al., 2016; Prather et al., 2012, 2021). Further, the experimental induction of disturbed sleep has also been shown to have a negative impact on vaccination outcomes (Lange et al., 2003, 2011; Spiegel et al., 2002), although not unequivocally (Benedict et al., 2012). Other indicators of potential sleep problems, such as shorter (<7hr) and longer sleep durations (>8–9hr) related to some immune outcomes such as higher C-reactive protein (CRP) levels indicating greater systemic inflammation (Dowd et al., 2011; Grandner et al., 2013). Similarly, needing to nap during the day, also appears to relate to inflammation such that individuals napping more in the day had higher CRP levels, especially among older participants (Leng et al., 2014) thus inflammation may explain why napping is sometimes associated with poor health outcomes (Mantua and Spencer, 2015). Across studies, one systematic review concluded that longer sleep and sleep disturbance related to higher inflammatory markers, specifically Interleukin-6 (IL-6) and CRP (Irwin et al., 2016). The relationship between sleep and immunity, and specifically antibody responses, is in part driven by circadian rhythms in the immune system relating to light/dark and rest/activity cycles. For example, in the blood many immune cell numbers peak during the rest phase whereas glucocorticoids and pro-inflammatory cytokines peak during active phases, i.e., during the day, likely to enable the host's immune system to respond to microbial threats more efficiently (Scheiermann et al., 2013). Recently, a study demonstrated that dendritic cells migrate from the skin to the draining lymph node in a time-of-day-dependent manner, influencing the immune response to vaccinations against Hepatitis A virus and SARS-CoV-2 (Ince et al., 2023).

The capacity of these health behaviours to significantly impact immunity, and specifically the antibody response to vaccination suggests that they would be important public health targets to improve immunity and immune-system related health outcomes. This is perhaps most important among groups particularly vulnerable to poorer immune function such as older adults. Exercise has been related to reducing a range of markers of immunesenescence or immune ageing (Kohut and Senchina, 2004) yet age moderates the antibody response to vaccination even among those who are physically active (Bohn-Goldbaum et al., 2022).

Thus far, most vaccination studies with a longitudinal follow-up have focused on initial or peak responses to vaccination and decline in antibody levels in the relatively short-term following vaccination, i.e., months rather than years. Less is known about the maintenance of antibody levels induced by natural infection and/or vaccination which may have occurred earlier on in life. These are antibodies developed in response to a prior vaccination as part of public health programmes, or prior infection (although not necessarily clinical disease) resulting from exposure to pathogens in the natural environment (“Types of Immunity to a Disease | CDC,” n.d.). Many diseases are combatted with vaccination at different points of the life course, however, maintaining protection in the form of immune memory and the ability to produce antibodies varies across individuals (Combadière et al., 2010). This is particularly crucial for the large and disproportionate population of older adults whose T-cell related antibody response against many pathogens declines early in immunesenescence (Pera et al., 2015). It is also a reason for repeated vaccinations among older adults beyond the mutation of pathogens and emergence of new variants.

If such health behaviours influence antibody maintenance, then interventions to improve them should help maintain protection against common infectious diseases. Many vaccination or antibody studies have focused on younger healthy adults or specific sub-samples of older people such as caregivers, where responses post-vaccination are compared with those measured at baseline and the magnitude of the vaccination response has been related to factors such as stress, social support and coping (for summaries see e.g., Burns et al., 2007; Glaser and Kiecolt-Glaser, 2005; Phillips, 2011). Further, in studies where time has elapsed since vaccination, and measuring baseline antibody levels and giving vaccines was not part of the study design, in those vaccinated over 12 months ago but not in the past year, antibody levels have still been shown to relate to psychosocial factors such as stress exposure, coping and psychological wellbeing (Burns et al., 2002a; Burns et al., 2002b). This suggests that the maintenance of antibodies is amenable to influence by psychosocial and/or behavioural factors, but again such studies have not focused on vulnerable groups such as older adults.

Consequently, the aim of the present study was to examine the association of specific key health behaviours (PA and sleep) with the maintenance of antibody levels following natural infection and/or vaccination in a secondary analysis of an existing dataset from a vaccination study in older adults (Phillips et al., 2006). It was unknown whether such factors would relate to antibody levels maintained over time rather than in response to vaccination. However, it was hypothesised that if any associations did emerge, they would be in the direction of higher PA and better sleep relating to higher antibody titres.

## 2 Method

### 2.1 Participants and design

This analysis is part of a larger longitudinal study of psychosocial factors and influenza vaccination antibody response in 184 older adults aged 65+ (104 women) recruited from five medical practices in Birmingham, UK, in autumn/winter 2003. Participants were excluded if they had a current acute illness e.g., a cold, a current immune suppressive disease such as blood cancer, or were taking immunosuppressive medication. Full design and participant details are given in the primary publication (Phillips et al., 2006). The present sample for analysis consisted of n = 104 individuals (60 women) who provided a blood sample at baseline where there was sufficient stored serum remaining for analysis of antibodies to a range of naturalistic pathogens/previous vaccinations. Participants also completed a questionnaire pack at baseline, 1 month, and 12 months. This included psychosocial measures as well as the health behaviours. Physical activity was measured at baseline and one month. Sleep was measured at 12 months. The sample were predominantly white (97%) and non-smokers (93%). The study was approved by the University of Birmingham Research Ethics Committee, and all participants provided written informed consent.

### 2.2 Questionnaires

Standard socio-demographic and clinical information was obtained at baseline, including age, current or previous occupation, height and weight, and presence of chronic medical conditions. Current/previous occupation was utilized as an index of socio-economic status using the Registrar General's Occupations; as the data were skewed, this was converted to a binary variable of manual versus non-manual occupation. Height and weight were used to calculate Body Mass Index (BMI) using the equation BMI = weight (kg)/height (m^2^).

#### 2.2.1 Health behaviours

In addition to the psychosocial questionnaires which formed the original study (Phillips et al., 2006), habitual PA was assessed at baseline and one month for the previous year or previous month, respectively, using questions adapted from the Whitehall II study (Marmot, Davey-Smith, Stansfield, Patel, North, Head, White, Brunner & Feeney, 1991). Participants were asked, on average how much time they spent in activities of light, moderate and vigorous exercise intensity (0, 1–2, 2–5, 6–8, 9–10, 11+ hours per week). The category scores (coded as 0,1,2,3,4, or 5), derived from the above were multiplied by a weighting of 1, 2, and 3 for light, moderate, and vigorous intensity activity respectively, and the products summed to yield a composite score.

At 12 months, in addition to the psychosocial questionnaires which formed the original study, participants also completed a more detailed sleep questionnaire abbreviated from the Pittsburgh Sleep Quality Index (PSQI) (Buysse et al., 1989) which asked about sleep over the past month including: bed time and getting up time, time taken to fall asleep (minutes), minutes of sleep lost due to waking up in the middle of the night, minutes of sleep lost due to waking up earlier than your usual time to get up, sleep quality over the past month (on a 4-point scale from very bad to very good), trouble staying awake, and napping during the day (both measured on 4-point scales: never, < once a week, 1–2 times a week, 3 or more times a week). This measure showed adequate internal consistency of alpha = .54, suggesting the individual items were not strongly associated and were better used as single measures. To match as far as possible with full PSQI components (Buysse et al., 1989), time in bed (hours) was calculated as the distance between bedtime and getting up time from questions one and two. Sleep duration was calculated as total time in bed minus minutes taken to fall asleep and minutes of sleep lost during the night minus minutes lost through waking early from questions one to five. Sleep efficiency was calculated as sleeping duration divided by time in bed (in minutes) * 100% (Schutte-Rodin et al., 2008). Subjective sleep quality was taken from the rating of sleep quality (question six). Sleep latency was reflected in question three: minutes taken to fall asleep and converted to a score from 0 to 3 (<15min, 16–30 min, 31–60 min, >60 min) (Buysse et al., 1989). This measure did not include a question about keeping up enough enthusiasm to get things done, sleep medication or ratings of frequency of different reasons for having trouble sleeping. Daytime dysfunction was captured through two separate questions: trouble staying awake during the day and naps during the day.

### 2.3 Blood samples and immunological assays

Venous blood was collected from an antecubital vein into two 7 ml plain tubes (BD Vacutainer, Meylan Cedex). The blood samples, which were allowed to clot for at least 1 h, were centrifuged at 3500 rpm for 5 min and the separated serum was frozen at −20 °C on the day of collection. Antibody assays were conducted on the baseline sample from the stored serum in summer 2007, in which time period and storage conditions they are still deemed stable (Hendriks et al., 2014; Michaut et al., 2014) The concentration of 19 anti-bacterial antibodies were quantified using a multiplexed Luminex assay, detailed previously (Whitelegg et al., 2012). IgG antibody levels were measured against 12 pneumococcal (Pn) serotypes, 4 meningococcal (Men) serotypes, Haemophilus influenzae type b (Hib) polysaccharide, and tetanus and diphtheria toxoids. Brief details of the assay process can be found elsewhere (Chicca et al., 2020) Fluorescent intensity was measured with a Luminex-100 instrument (Luminex Corp, TX, USA). Data acquisition was performed on acquisition software (Bio-Plex Manager software version 4 BioRad Laboratories, CA., USA) to generate serotype antibody concentrations. The protective thresholds for IgG were: 0.35 μg/ml for Pn serotypes (“WHO/Health Canada Consultation on Serological Criteria for Evaluation and Licensing of New Pneumococcal Vaccines,” n.d.), 2 μg/ml for Men serotypes (Peltola et al., 1977), 1 μg/ml for Hib polysaccharide (Kayhty et al., 1983), and 0.1 IU/ml for diphtheria and tetanus (Plotkin, 2001). Streptococcus pneumoniae capsular polysaccharides were obtained from the American Type Culture Collection (Manassas, Virginia, USA), Tetanus toxoid was obtained from Quadratech (Epsom, UK), Neisseria meningitidis and Hib capsular polysaccharides, and diphtheria toxoid were from the National Institute for Biological Standards and Control (Potters Bar, UK).

### 2.4 Data reduction and analysis

Statistical analyses were conducted with IBM SPSS version 28. Associations between socio-demographics, health behaviour variables and antibody titres were analysed initially using correlations and independent t-tests where appropriate. Due to the skewed distribution, antibody levels were subject to log_10_ transformation. The basic immunological data are also reported in terms of the number of participants showing a protective antibody titre taken from the criteria described above. For the main analyses, correlations were run to explore any potential associations between health behaviours (PA at baseline and one month, and sleep at 12 months) and antibodies. Where health behaviour variables were ordinal rather than interval or ratio (sleep quality score, sleep latency score, sleep dysfunction, naps), non-parametric Pearson's correlations were conducted. To assess potential confounding, associations between socio-demographics and both antibody and health behaviour variables were explored using correlations, t-tests and chi-square as appropriate to the type of variable. Significant correlations between health behaviour variables and antibodies from the main analysis were then repeated adjusting for any socio-demographic variables that significantly related to either antibodies or health behaviours. Due to multiple analyses, the findings from the above analyses had the Benjamini-Hochberg correction for false discoveries applied (Benjamini and Hochberg, 1995), with a false discovery acceptance rate set at 20%. This rate of 0.2 was selected as there is a low cost to a false positive in the present analyses given this is the first attempt to look at PA/sleep and the maintenance of antibodies.

For clinical sensitivity analysis and illustrative purposes, any significant associations arising from the main analyses were repeated in a series of ANOVAs (or non-parametric Kruskal-Wallis tests) using the binary cut-off criterion for protection for each pathogen as the fixed factor. For completeness, any significant ANOVAs were rerun as ANCOVAs (or non-parametric equivalent) adjusting for potential confounders as above.

## 3 Results

### 3.1 Questionnaire data

Participant socio-demographics and questionnaire scores are shown in Table 1. The sample was nearly two thirds female, mainly white and non-smokers. Half had manual versus non-manual occupational status, just under two thirds reported a chronic illness (mainly high blood pressure or arthritis) and just over two thirds reported taking continuous medication.

**Table 1:** Descriptive statistics for socio-demographic variables and health behaviours.

Variable	N	Mean/n	SD/%	Median	Mode	Range
Age (years)	103	73.3	5.98	–	–	–
Sex - female	104	60	58	–	–	–
Ethnicity – non-white	100	3	3	–	–	–
Smoker - yes	97	7	7	–	–	–
Occupation – non-manual	94	47	51	–	–	–
Chronic illness - yes	99	64	65	–	–	–
Taking medication - yes	97	72	74	–	–	–
PA score baseline	90	3.3	2.79	–	–	–
PA score 1 month	98	3.6	2.90	–	–	–
Time in bed (hr)	104	8.3	1.06	–	–	–
Sleep duration (hr) and score (0–3)	92	7.4	1.25	0	0	1
Sleep efficiency (%)	92	88.8	9.60	–	–	–
Sleep latency (mins) and score (0–3)^a^	100	24.8	23.40	0	0	3
Sleep quality (0–3)^a^	104	–	–	0	0	3
Trouble staying awake (0–3)^a^	103	–	–	0	0	2
Daytime napping (0–3)^a^	104	–	–	2	2	3

a a lower value indicates less time to fall asleep, lower quality, less trouble staying awake in the day, less frequent naps.

### 3.2 Antibody data

Descriptive statistics for participants’ raw and logged antibody data are shown in Table 2. Age was only significantly associated with higher titres against Pn5 (p = .02) and more naps per day (p = .02); number of previous influenza vaccinations was not related to antibodies or any PA or sleep variable. Sex was not related to antibody titres, PA or sleep, with the exceptions that women had longer sleep latency (p = .02) and took fewer naps (p = .04) than men. Finally, the presence of chronic illness only significantly related to longer sleep latency (p = .04). Hence these variables (age, sex, chronic illness) were not adjusted for in later analyses, except where initial significant associations emerged for Pn5, and/or naps, or sleep latency.

**Table 2:** Average antibody data (n = 104).

Pathogen	Raw Mean (μg/ml)	SD	Log_10_ Mean
Pneumococcal Type 1	0.7	1.75	−.88
Type 3	1.4	2.80	−.45
Type 4	0.4	1.01	−.71
Type 5	2.5	3.14	.02
Type 6b	1.8	2.94	−.16
Type 7f	1.7	2.25	−.05
Type 9v	2.0	4.14	−.26
Type 14	3.1	5.21	−.03
Type 18c	4.2	4.78	.17
Type 19a	2.7	3.17	.09
Type 19f	1.4	1.54	<.01
Type 23f	1.8	2.59	−.26
Meningococcal A	2.7	2.41	.30
Meningococcal C	0.4	1.54	−1.44
Meningococcal W	0.2	0.62	−1.12
Meningococcal Y	1.2	1.70	−.13
Haemophilus B	0.7	1.55	−.64
Diphtheria (IU/ml)	0.04	0.18	−2.30
Tetanus (IU/ml)	2.0	2.88	−.35

### 3.3 Associations between PA and logged antibody titres

Correlations revealed significant positive associations between PA score at baseline and Pn14, Pn18c, Pn23f, and MenY. PA score at one month significantly related to six Pn serotypes (Pn1, Pn14, Pn18c, Pn19a, Pn19f, Pn23f). Significant associations between PA scores and several serotypes are shown in Table 3. All associations were positive, indicating that a higher PA score related to higher antibody levels. Following application of the Benjamini-Hochberg correction, the findings for baseline PA and Pn18 and Pn23f were no longer significant. However, for PA at one month all of the original findings remained significant, and there were also now significant positive associations for Pn3, Pn6b, MenY and Hib.

**Table 3:** Correlations between physical activity score and log_10_ antibody titres.

	Pn1	Pn3	Pn4	Pn5	Pn6b	Pn7f	Pn9v	Pn14	Pn18c	Pn19a	Pn19f	Pn23f	MenA	MenC	MenW 135	MenY	Tet.	Dip.	Hib
PA baseline	.20	.13	.19	.06	.10	.13	.02	.25*	.22*	.14	.09	.21*	.06	.12	.18	.26**	−.11	−.05	.05
PA 1m	.25*	.18	.07	.10	.18	.15	.11	.24*	.34***	.27**	.26*	.29**	.08	.08	.14	.17	.01	−.05	.17

Note: *p < .05, **p < .01, ***p < .001. For PA baseline n = 90; for PA one month n = 98. Tet. = tetanus, Dip. = diphtheria. Hib = Haemophilus influenzae type b.

### 3.4 Associations between sleep variables and logged antibody titres

Correlations revealed significant associations between several sleep variables and antibody levels. These were between: time in bed and Pn4, sleep duration and Pn19f, and sleep efficiency and Pn5. Non-parametric correlations were significant between sleep quality and Pn19f, sleep latency and Pn5, trouble staying awake during the day and Pn19a, Pn19f, MenA and MenY, and finally, daytime napping and Pn6b, Pn19f, Pn23f, and MenY. As can be seen from Table 4, longer time in bed and longer sleep duration related to higher antibodies against Pn4 and Pn19f, respectively. Higher sleep dysfunction measured as trouble staying awake in the day related to lower antibodies against two Pn and two Men serotypes, and more frequent daytime naps related to lower Pn6b, Pn19f, Pn23f, and MenY antibody levels. Surprisingly, higher sleep efficiency and shorter sleep latency related to lower antibodies against Pn5, and higher sleep quality related to lower Pn19f antibodies.

**Table 4:** Correlations between sleep variables and log10 antibody titres.

	n	Pn1	Pn3	Pn4	Pn5	Pn6b	Pn7f	Pn9v	Pn14	Pn18c	Pn19a	Pn19f	Pn23f	MenA	MenC	MenW 135	MenY	Tet.	Dip.	Hib
Time in bed	104	.18	.08	.20*	.13	.05	.02	.12	.02	.15	.11	.15	.16	.09	.19	.04	.10	.06	.11	.13
Duration	92	.09	.02	.03	−.02	.01	.01	.11	−.06	.06	.01	.21*	.12	.06	.03	−.05	.03	.06	.16	.09
Efficiency	92	−.14	−.04	−.18	−.24*	−.01	−.01	−.07	−.09	−.08	−.11	.13	−.03	−.06	−.08	−.14	−.07	−.00	.10	−.04
Latency^#^	100	.11	.02	.12	−.22*	−.05	−.03	−.01	−.07	.03	.04	−.12	−.08	.03	−.00	−.05	.06	.05	−.04	−.13
Quality^#^	104	−.02	−.06	−.08	.02	-.-8	−.16	−.12	−.02	−.04	−.14	−.32**	−.04	−.13	−.01	−.11	−.08	.06	−.16	−.08
Dysfunction	103	−.17	−.03	−.07	−.10	−.06	−.10	−.14	.03	−.09	−.21*	−.23*	−.15	−.29**	−.04	−.12	−.25*	.04	−.06	−.09
Napping	104	−.04	−.04	−.06	−.07	−.24*	−.04	−.09	.16	−.12	−.03	−.29**	−.24*	−.15	−.15	−.18	−.22*	.01	−.02	−.09

Note: *p < .05, **p < .01, ***p < .001. Dysfunction = trouble staying awake. Tet. = tetanus, Dip. = diphtheria. Hib = Haemophilus influenzae type b. ^#^lower value indicates shorter time to fall asleep, and lower quality.

Adjustment for significant socio-demographic variables meant that significant associations including Pn5 were adjusted for age, and associations with naps were adjusted for age and sex. Consequently, partial correlations revealed that more frequent daytime napping still related to lower antibodies against Pn6b (p = .02), Pn19f (p = .003), Pn23f (p = .02), and MenY (p = .01) after adjustment for sex. However, higher sleep efficiency was now only marginally significantly associated with Pn5 (p = .052), and greater sleep latency no longer related to lower antibodies against Pn5 after adjusting for age, sex, and chronic illness (p = .07).

Following the Benjamini-Hochberg correction of initial analyses including those rerun adjusting for potential confounders, the findings for time in bed, sleep duration, sleep quality, trouble staying awake in the day, and daytime napping all remained significant, with the non-significant findings for sleep efficacy and latency remaining non-significant.

### 3.5 Sensitivity analyses

**Fig. 1:**
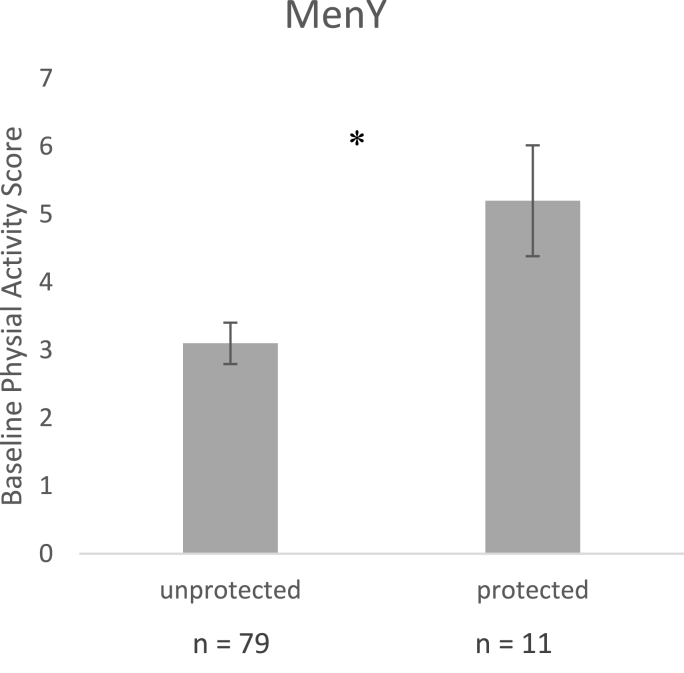
Differences in PA score at baseline between those with seroprotection and those not protected against MenY (n = 90).

**Fig. 2:**
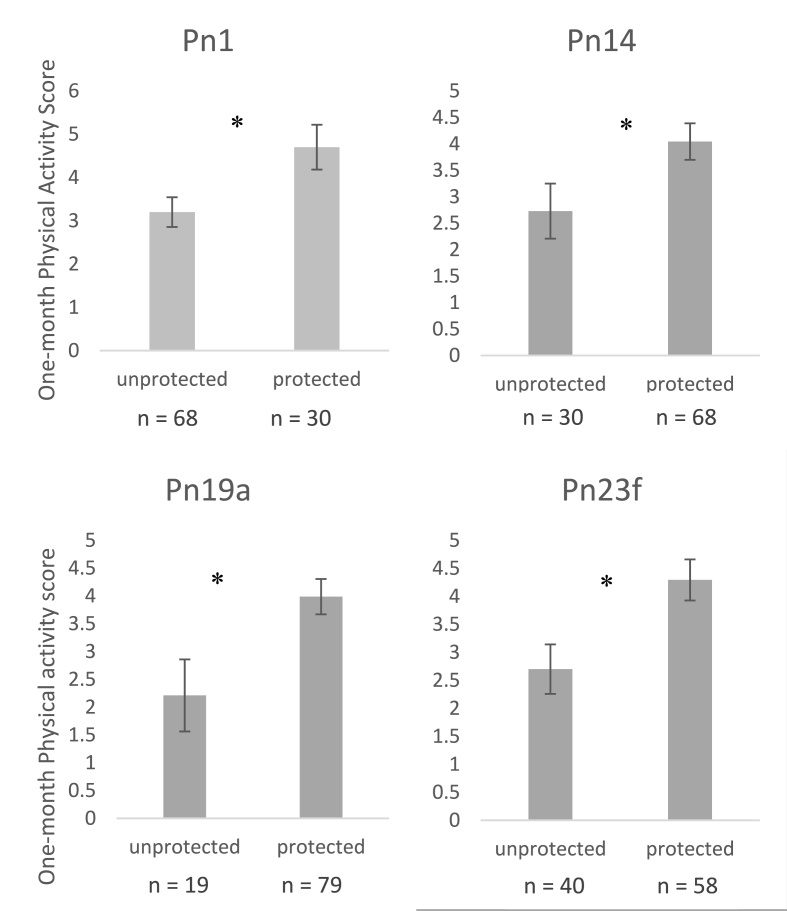
Differences in PA score at one month between those with seroprotection and those not protected against Pn serotypes (n = 98).

Binary variables for each pathogen were created based on the seroprotection cut-off criterion for each applied to the raw antibody titres. A series of ANOVAs with each binary variable as a fixed factor were then run for any significant associations that arose in the primary analyses above, to see whether health behaviours related to being seroprotected or not. Significant group differences are shown in Fig. 1, Fig. 2 for PA, such that only seroprotection against MenY differed on PA score at baseline (p = .02), but seroprotection against Pn1, 14, 18, and 23f did not (p = .16, .14, 0.31, and 0.09, respectively). For PA at one month (Fig. 2), four of the original six Pn serotypes remained significant such that those who reported higher PA were more likely to be seroprotected against Pn1 (p = .02), Pn14 (p = .04), Pn19a (p = .02) and Pn23f (p = .007), but not Pn18c or Pn19f (p = .06, and .12, respectively).

**Fig. 3:**
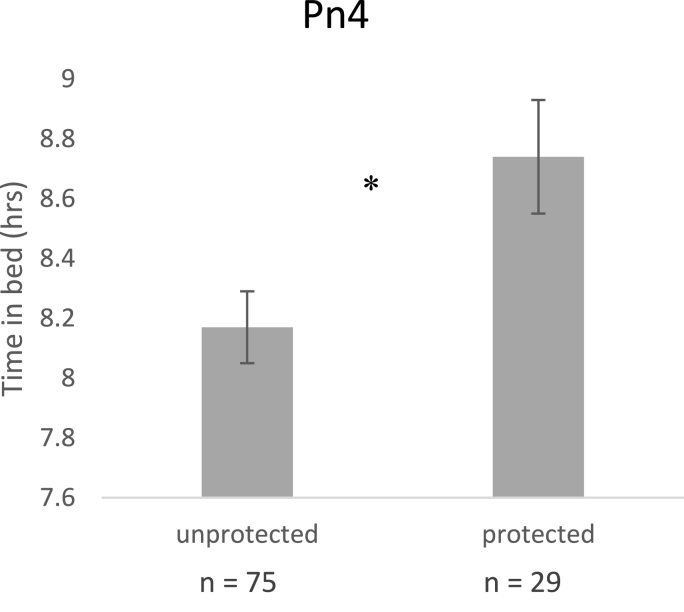
Differences in time in bed between those with sero-protection and those not protected (n = 104).

From ANOVAs, significant differences in seroprotection for sleep variables are shown in Fig. 3, such that those who spent longer time in bed were still more likely to be seroprotected against Pn4 (p = .01) but sleep duration no longer related to Pn19f (p = .88) and sleep efficiency no longer related to Pn5 antibodies (p = .06). From non-parametric tests, trouble staying awake significantly differed across those protected and unprotected against Pn19a (p < .001) and MenA (p = .02), but not Pn19f (p = .48), or MenY (p = .22). Frequency of daytime napping significantly differed across seroprotection to Pn6b (p = .02), but not Pn19f (p = .43) or Pn23f (p = .34) or MenY (p = .46). However, sleep quality no longer related to Pn19f (p = .06); and sleep latency no longer related to Pn5 (p = .30). For completion, analyses were repeated adjusting for potential confounders as covariates where appropriate. For daytime napping and seroprotection with adjustment for age and sex as covariates, which showed the same pattern of results as above such that significant differences in napping were found for Pn6b (p = .01), but not Pn19f (p = .31), Pn23f (p = .36) or MenY (p = .33). For sleep latency and Pn5 seroprotection with adjustment for age, sex and chronic illness, similar to above there was no significant difference in sleep latency between those protected or not (p = .71).

## 4 Discussion

In age-adjusted analyses, higher PA at baseline significantly related to higher antibody levels against three Pn serotypes and MenY; and higher PA at one month related to higher antibody levels against six Pn serotypes. Longer time in bed related to higher antibody titres against Pn4, and longer sleep duration related to higher antibody titres against Pn19f. Less trouble staying awake during the day related to higher antibodies against two Pn and two Men strains, and less daytime napping related to higher antibodies against Pn6b, Pn19f, Pn23f and MenY. Somewhat surprisingly, lower sleep quality related to higher antibody titres against Pn19f. Using the clinical criterion of seroprotection as the outcome measure revealed slightly fewer significant effects but the same direction of effects for PA benefitting antibody protection. For sleep variables, only time in bed remained significant in binary analyses with Pn4 and Pn5, respectively; sleep duration, efficiency, latency, and quality were no longer significant.

This study shows for the first time that habitual regular PA is related to the maintenance of immunity following naturalistic infection and/or prior vaccination among older adults. This adds to what is known about how health behaviours such as habitual PA or PA interventions relate to the peak antibody response formed following vaccination and initial decline over time observed in some studies e.g., (Chastin et al., 2021; Felismino et al., 2021; Zheng et al., 2015). However, such PA effects were not found in all studies, e.g. (Long et al., 2013; Hayney et al., 2014), which may reflect differences in population sampled, or the PA intervention used in terms of mode, duration and intensity. It is important to note that this association between antibody titres and habitual PA is different to associations observed between antibody responses and the influence of an acute bout of exercise proximal to the time of vaccination, as shown in e.g., (Edwards and Booy, 2013). Further, most studies examining habitual PA or PA interventions have focused on the response to influenza vaccination as opposed to a range of vaccines, however, this has been useful in distinguishing the positive impact of habitual PA in combination with acute exercise effects (Bohn-Goldbaum et al., 2022). Future longitudinal studies of longer-term monitoring of PA are needed in the context of vaccination, including potentially comparing different modes, frequency, duration, or intensities of PA intervention on effectiveness of antibody maintenance in a range of ages and to a range of vaccinations to provide more conclusive answers.

Overall, moderate-to-vigorous physical exercise relates to a higher resilience against infectious diseases in previous epidemiological or habitual PA level group comparison studies (Hamer et al., 2019; Zheng et al., 2015). Further, one meta-analysis of RCTs examining PA interventions showed that PA enhanced the potency of vaccination (Chastin et al., 2021). Effects for PA on antibody responses to vaccination have also been shown to be more prominent in older adults (Bohn-Goldbaum et al., 2022). These findings are somewhat in line with the findings of the present study, although it should be acknowledged that the present study mainly found associations for various pneumococcal serotypes whereas one meta-analysis referred to influenza antibodies only (Bohn-Goldbaum et al., 2022), and the other meta-analysis also reported effects mainly for influenza with the exception of two studies (Chastin et al., 2021) of which the pneumococcal vaccination study was not significant (Long et al., 2013). Further, a recent systematic review revealed that higher PA levels related to higher influenza vaccine antibodies compared to no PA but had no association with pneumococcal antibodies across cross-sectional and intervention studies (Dinas et al., 2022). This contrasts with the current findings which were relatively robust for Pn effects even following adjustment for covariates. This may be explained by the positive impact of exercise on T-regulatory cells (Proschinger et al., 2021) which may be more involved in the response to thymus-dependent antigens such as influenza than thymus-independent pathogens such as pneumococcal polysaccharides. However, the meta-analysis examined antibodies in response to vaccination only (Dinas et al., 2022), so is not directly comparable to the present antibody levels resulting from naturalistic exposure *or* prior vaccination. Potentially the factors which influence peak response to vaccination may differ from those which influence antibody maintenance or reflect that influenza vaccination studies are more common than pneumococcal studies. Further, this systematic review of both PA interventions and measurement of habitual PA further acknowledges the heterogeneity of available evidence regarding populations, types of vaccination, follow-up duration post-vaccination, and PA measurements, which perhaps explains the contrasting findings and opens future research avenues such as the need for research on the impact of health behaviours on longer-term maintenance of antibodies following vaccination.

Certain Pn antibody levels were significantly positively correlated with time in bed and sleep duration. This adds to the evidence suggesting sufficient sleep is critical to enhancing the immune system (Bryant et al., 2004) and immune response after vaccination (Lange et al., 2011; Rayatdoost et al., 2022). For example, experimental research shows that lack of sleep impairs immune function, as seen by decreased activity of natural killer cells, suppressed interleukin-2 production, and elevated levels of circulating pro-inflammatory cytokines (Irwin et al., 2006; Opp, 2009; Vgontzas et al., 2004). This is not surprising given that interference with the natural circadian rhythms of the immune system results in shifts in immune system activity through changes in cell and humoral immune signalling as well as glucocorticoid release. Such signalling patterns are driven by the cyclic expression of clock genes regulated by the suprachiasmatic nucleus of the hypothalamus (Harrington, 2010) which is why several immune-mediated diseases are linked to disrupted circadian rhythms (Scheiermann et al., 2013). Interestingly, in one study, time in bed was related in a U-shaped association with CRP but night-time sleep duration was not related to CRP levels (Leng et al., 2014). However, in another study, sleep duration related to CRP in a U-shaped association, but sleep quality did not emerge as a significant predictor of CRP (Dowd et al., 2011). The associations between longer sleep and inflammation discovered by prior research may in fact represent the effects of longer time in bed relating to poor overall health, rather than longer sleep *per se*; as epidemiological studies have used a variety of methods to define sleep duration (Leng et al., 2014), or may even reflect both sleep-related and circadian-related impacts on inflammation. In a similar pattern and in support of the present findings, a prospective study of 56,953 females from the Nurses' Health Study II cohort (aged 37–57 years) found that when compared to 8-h sleepers, both shorter and longer sleep durations and perceived inadequate sleep were associated with an increased pneumonia risk (Patel et al., 2012).

In terms of daytime dysfunction, more daytime naps related to significantly lower antibody titres against several pathogens, which remained robust following adjustment for covariates. This direction of effects for these variables is perhaps not unexpected given that the need to nap during the day may relate to not sleeping well at night, reflecting the same direction of effects for shorter time in bed and sleep duration in the present study. Although daytime napping may have several health benefits, frequent and prolonged naps may be associated with higher morbidity and mortality, especially in older adults (Dhand and Sohal, 2006). Taking excessive daytime naps is associated with higher respiratory disease incidence risk (Leng et al., 2016) and higher CRP levels, especially in older participants, suggesting that the need for naps during the day may be related to inflammation (Leng et al., 2014). This supports the growing theory that inflammation mediates the association between excessive daytime napping and adverse health outcomes (Mantua and Spencer, 2017). Replication of the present findings and mechanistic studies may help confirm whether this is also the case for immune outcomes such as antibody levels or whether a different mechanism is involved. The pro-inflammatory state during nocturnal sleep can be regarded as positive and beneficial for adaptive immunity such as antibody production, however, inflammation during the day is associated with sickness behaviours including fatigue, thus similarly might be detrimental for adaptive immunity (Besedovsky et al., 2012; Petrovsky, 2001).

A similar pattern of effect emerged for trouble staying awake in the day relating to lower antibody levels. This might be explained through the associations with sleep duration and daytime napping such that those with adequate sleep and less need for naps would also be those less likely to have difficulty staying awake in the daytime. Indeed, these variables were significantly correlated in the present study. Thus, the inflammation explanation for napping effects above applies to this variable also.

A surprising direction of findings emerged for sleep efficiency, sleep latency, and sleep quality, such that lower efficiency, longer latency, and lower quality sleep were associated with higher antibody levels. This is the reverse of what might be expected in relation to sleep. However, the present inverse findings should be considered carefully for several reasons. First, associations only emerged for Pn5 for two of these (efficiency and latency) and for Pn19f only for sleep quality, rather than to a range of pathogens. Second, unlike the variables discussed above, effects for sleep efficiency and latency were no longer significant following adjustment for significant socio-demographics. Third, although the sleep quality effect did not require adjustment for confounders, it is notable that there was little variance in sleep quality reporting in that only one participant reported very good sleep, and 83% reported very bad sleep. Fourth, the findings for sleep quality, efficiency or latency were not robust in the binary analyses for seroprotection. Fifth, the antibody levels against Pn19f are higher compared to other serotypes with 93% of participants having levels above the WHO seroprotective threshold. Finally, others have shown that sleep deprivation may only be a risk factor for lowered immunity, at least to influenza, in the short term (Benedict et al., 2012; Spiegel et al., 2002), and it is not known how long ago the present participants were exposed to the pathogens measured here.

The present study typically showed significant effects across health behaviours for pneumococcal and meningitis A and Y serotypes. Older adults are unlikely to have received childhood or adolescent vaccination against meningococcal serotypes or Hib, diphtheria, or tetanus due to when these programmes began (UKHSA, 2004) thus, they would be dependent on naturalistic exposure or opportunistic vaccination due to increased risk, e.g., tetanus due to a wound (“Tetanus - NHS,” n.d.). However, the pneumococcal polysaccharide vaccines started to be given routinely in 2003, the year of the present study, for all older adults aged 65+ years and those with comorbidities in the UK. Coverage levels varied by GP surgery policy and were estimated at 29–36% in England between 1991 and 2003 (Noakes et al., 2006) but given this was a sample already attending their GP for an influenza vaccination it is possible that uptake was higher. Perhaps more convincingly, the pattern of findings across pneumococcal and meningococcal serotypes may reflect development of naturalistic protection due to exposure to children, e.g., grandchildren who at the time of the study were not vaccinated to pneumococcus and likely to carry many of these serotypes as commensal organisms. All the serotypes tested in the present study were those contained in the childhood conjugate pneumococcal vaccine prevnar 13, and adult pneumococcal disease incidence has fallen since the introduction in 2006 of the 7-valent and in 2010 the 13-valent vaccine programmes in infants, while incidence of serotypes not contained in those vaccines have increased (Ciruela et al., 2019) although there is some evidence that this protective effect of herd immunity may be limited for some serotypes (Van Der Linden et al., 2019). Similarly, children receiving conjugate meningococcal vaccines (UKHSA, 2004), will reduce exposure of adults to those bacteria.

An alternative reason for the present pattern of findings may lie in the differences in the response to each pathogen measured. For example, as mentioned above, most of the sample were seroprotected against Pn19f, whereas only three, one and six percent were protected against MenC, MenW135, and diphtheria, respectively. This lack of variation does not allow space for any associations with health behaviours as it represents a floor/ceiling effect. However, this explanation is less plausible for the lack of any associations for Pn3, Pn6b, Pn7f, Pn9v, Hib or Tetanus where there were between 18 and 84 participants seroprotected, depending on the specific pathogen. It is perhaps important to note, however, that although not significant, many of the effects for PA at baseline and one month were related to other Pn and Men serotypes in the same direction as the significant findings presented above, and indeed several of these associations were trends (p = .06 - 0.1). This was also the case for sleep variables such as duration, albeit to a lesser extent. This suggests that the availability of prior pneumococcal vaccination and/or naturalistic exposure, particularly to young children, may underlie the present pattern of effects. However, this would require considerable further investigation in a sample tracking from multiple vaccinations and/or disease exposure longitudinally to fully elucidate the reason.

### 4.1 Limitations

The present study has limitations. First, short or adapted versions of measures were used to assess PA and sleep, rather than more standardized accepted measures such as the IPAQ (Craig et al., 2003) or PSQI (Buysse et al., 1989). However, similar totals for PA intensity by frequency and sleep measures e.g., duration, quality and efficiency were able to be derived from the included measures. It should be acknowledged though that even with deriving sleep measures in a similar way to those from the PSQI, there is debate in the literature surrounding the calculation of sleep efficiency from total time in bed rather than intended sleep time (Reed and Sacco, 2016). Further, other sleep-related variables which might be expected to contribute to poor sleep quality such as sleep apnea or snoring were not assessed in this study, and would be worth including in future research. Second, objective assessment of PA and sleep using accelerometry and electroencephalography devices would be the gold standard for measurement rather than self-report and provide the opportunity to examine the relationship between sleep architecture and immune function. For example, slow wave sleep appears to be particularly important for the extravasation of T-cells to the lymph nodes and the formation of immune memory via T-helper cells and antibody numbers (Besedovsky et al., 2012). However, this is time-consuming and costly in larger samples and the present study was a secondary data analysis so such measures could not be added. Third, it should be acknowledged that the longer questionnaire on sleep variables was not measured at the time of antibody assessment but 12 months later, although PA was measured at baseline, due to attempt to reduce participant questionnaire burden. However, although some sleep variables change across the lifespan, this is less the case for sleep duration than sleep timing (Evans et al., 2021) and in later life it is likely that only small changes occur over the course of a year given trends across later life (Wallace et al., 2022). In addition, given this study was not a health behaviour intervention, it was not expected that there would be any systematic change across participants in PA between baseline and one month. Finally, it was not possible to ascertain whether participants’ antibodies were from naturalistic exposure or vaccination against the pathogens or how long ago this was, making it difficult to assess the duration of antibody maintenance. However, measuring antibodies against a range of common pathogens, including those unlikely to have arisen from naturalistic exposure gives an insight into the general maintenance of antibodies among older adults across different types of antibody response (thymus dependent/independent etc.). The overall findings presented here should, of course, be considered as preliminary given that the design is correlational, presenting only associations between PA/sleep scores and antibody levels. Thus, causality cannot be inferred and experimental and/or intervention designs would be necessary to gain insights into potential mechanisms and directions of effect. However, these findings suggest a potential direction for future research.

### 4.2 Future directions

Numerous factors can affect the immune response following vaccination and/or naturalistic exposure, including intrinsic host factors (such as age, sex, BMI, genetics, and comorbidities), extrinsic factors (such as co-infection), behavioural factors (such as PA, stress, sleep, smoking, and alcohol consumption), nutritional factors (such as nutritional status and dietary intake), environmental factors (such as rural versus urban environment, season, and geographic location), vaccine factors (such as type, adjuvants, dose, and booster dose) and administration factors (such as vaccination route and time of day) (Zimmermann and Curtis, 2019) as well as the availability and time of emergence of vaccination programmes as discussed above. However, now that vaccination programmes for older adults are standard, and comprehensive childhood vaccinations have been available and utilized for 20–80 years (UKHSA, 2004), there is plenty of potential to modify behaviours with the potential to influence antibody maintenance over time in older adults today. Some elements can be more easily modified to increase vaccine effectiveness and antibody longevity, such as getting enough PA and sleep. The present findings add to the consensus that regular PA and good sleep significantly contribute to healthy ageing (World Health Organization, 2015) and suggest that interventions aimed at increasing PA and improving sleep quality may be beneficial for older adults to maintain immunity against common infectious diseases. Additionally, healthcare providers may want to consider that napping may indicate underlying dysfunction even when working with older adults. As suggested above, further research would be needed to see whether these findings replicate to PA and sleep measured through objective means and to further explore the mechanisms underlying these relationships such as inflammatory markers and/or time since vaccination in longitudinal studies tracking participants from initial exposure.

## 5 Conclusion

In conclusion, this study aimed to explore the relationship between self-reported PA and sleep and antibody levels against a range of common infectious diseases in older adults aged 65+ years. Higher PA measured on two occasions was related to higher antibody levels against pneumococcal serotypes. Longer time in bed, longer sleep, less trouble staying awake in the day and less daytime napping were related to higher antibody titres against specific serotypes of pneumococus and meningococcus, although findings for sleep were less robust in analyses of seroprotection. Taken together these findings extend the existing literature demonstrating associations between PA and sleep and peak antibody response to vaccination. This suggests that promoting PA and healthy sleep habits may be an important strategy to maintain immunity in later life and reduce the risk of infection in older adults. However, these findings are preliminary and future research should seek to utilise objective measures and tracking of antibody levels from initial exposure/vaccination to examine mechanisms and the long-term impact of these health behaviours on antibody maintenance.

## Declaration of competing interest

The authors declare that they have no known competing financial interests or personal relationships that could have appeared to influence the work reported in this paper.

## Data Availability

The data that has been used is confidential.
